# The role of routine laboratory tests after unilateral total knee arthroplasty

**DOI:** 10.1186/s12891-022-05509-0

**Published:** 2022-06-10

**Authors:** An-an Li, Yu Zhang, Hao Zhang, Mei-ying Yan, Shi-ning Xiao, Nan-shan Zhong, Xin-hua Long, Shi-jiang Wang, Yang Zhou

**Affiliations:** 1 Department of Orthopedics, The First Affiliated Hospital of Nanchang University, Jiangxi, People’s Republic of China; 2 Medical Innovation Center, The First Affiliated Hospital of Nanchang University, Jiangxi, People’s Republic of China; 3 The Fourth Affiliated Hospital of Nanchang University, Jiangxi, People’s Republic of China; 4 Department of Radiology, Nanchang University Second Affiliated Hospital, Jiangxi, China; 5 Department of Emergency, The First Affiliated Hospital of Nanchang University, Jiangxi, People’s Republic of China

**Keywords:** Clinical intervention, Postoperative laboratory tests, Risk factor, Unilateral total knee arthroplasty

## Abstract

**Background:**

Recent studies suggest that routine laboratory tests are not required within 1 day after partial knee arthroplasty. In this study, we evaluated the utility of routine postoperative laboratory tests after initial unilateral total knee arthroplasty (TKA) in an Asian population. In addition, we explored risk factors associated with abnormal test results.

**Methods:**

Clinical data of patients who underwent original unilateral TKA between 2015 and 2020 were retrospectively analyzed. Patient characteristics and laboratory test results were recorded. Multivariate binary logistic regression analysis was performed to identify risk factors associated with 3 abnormal laboratory results.

**Results:**

A total of 713 patients, who underwent relevant laboratory tests within 3 days of TKA surgery, were enrolled. Among them, 8.1%, 9.9%, and 3.4% patients with anemia, hypoalbuminemia, and abnormal serum potassium levels required clinical intervention after surgery. Binary logistic regression analysis revealed that preoperative hemoglobin levels, estimated blood loss, and age were independent risk factors of postoperative blood transfusion in TKA patients. On the other hand, preoperative albumin levels, intraoperative blood loss, and operation time were risk factors associated with postoperative albumin supplementation. In addition, lower body mass index (BMI) and preoperative hypokalemia were potential risk factors of postoperative potassium supplementation.

**Conclusion:**

Considering that more than 90% of abnormal postoperative laboratory tests do not require clinical intervention, we believe that routine laboratory tests after surgery have little significance in patients with primary unilateral TKA. However, postoperative laboratory testing is necessary for patients with established risk factors.

**Supplementary Information:**

The online version contains supplementary material available at 10.1186/s12891-022-05509-0.

## Introduction

Total knee arthroplasty (TKA) is one of the most common surgery, and the mainstay treatment for end-stage knee osteoarthritis [1]. The TKA operation not only restores knee joint function but also reduces pain [2]. The increase in the aging population has resulted in a large number of TKA.

Laboratory tests have been traditionally used for diagnostic purposes to guide clinical decision-making. Blood tests after surgery are routinely used, especially after major orthopedic surgeries [3], such as TKA. Generally, postoperative laboratory tests are performed to prevent the omission of critical clinical details and potentially serious complications [4]. In the past decade, the concept of fast recovery and significant improvement of perioperative care pathways has significantly shortened the length of hospital stays and incidence of postoperative complications among patients [5, 6]. Previous studies have shown that the widespread use of tranexamic acid has significantly reduced the risk of blood loss and the rate of blood transfusion [7, 8]. Therefore, many scholars have questioned whether it is necessary to perform routine laboratory tests for patients without uncomplications. Some clinicians hold the view that there is no need for routine laboratory blood tests in patients without risk factors after surgery, such as those who undergo total hip replacement [4, 9], shoulder replacement [10], and partial knee arthroplasty [11]. However, a handful of researches have reported the need for routine laboratory testing in patients after TKA surgery [3].

In this study, we aimed to reevaluate the need for routine postoperative laboratory testing in patients who underwent primary elective unilateral TKA surgery. In addition, we analyzed the association of relevant clinical parameters with abnormal postoperative laboratory test results to identify risk factors associated with abnormal test results requiring clinical intervention.

## Methods

### Study subjects

Patients who underwent TKA surgery at our hospital between 2015 and 2020 were enrolled. Patients undergoing bilateral total knee arthroplasty, as well as those with infections, malignant tumors, and rheumatoid osteoarthritis, were excluded.

### Date collection

The patient’s demographic and clinical characteristics including sex, age, body mass index, and preoperative comorbidities were recorded. Surgical factors, such as intraoperative blood loss, and operative time, preoperative and postoperative laboratory parameters, including liver function, renal function, electrolytes, and inflammatory markers, as well as incidence of abnormal postoperative laboratory markers and related interventions, such as blood transfusion, albumin supplementation, and potassium supplementation were recorded. The risk factors of abnormal postoperative laboratory indicators requiring intervention were also obtained. The reference ranges of all laboratory test indicators in this study are listed in Table 1. The basic requirements for clinical intervention for abnormal laboratory indicators after surgery [12] are as follows: hemoglobin < 70 g/L or symptomatic anemia with a hemoglobin level of < 100 g/L, albumin < 30 g/L, serum potassium < 3.5 mmol/L.

**Table 1:** The normal reference ranges for laboratory values

Laboratory Tests	Reference range
Routine blood test	
Hemoglobin (g/L)	130–175
Platelets (*10^9/L)	125–350
Liver function test	
Alanine aminotransferase (IU/L)	9–50
Aspartate aminotransferase (IU/L)	15–40
Albumin (g/L)	40–55
Renal function test	
Creatinine (umol/L)	57–97
Blood urea nitrogen (mmol/L)	3.1–8.0
Inflammation indicators test	
Erythrocyte sedimentation rate(mm/h)	0–20
C-reactive protein(mg/l)	0–8
Electrolytes	
Serum sodium (mmol/L)	137–147
Serum potassium serum potassium (mmol/L)	3.5–5.3
Serum calcium (mmol/L)	2.11–2.52

### Decision to intervene

For patients with abnormal hemoglobin (Hb) i.e., Hb > 100 g/L, no clinical intervention was administered. For patient with Hb < 70 g/L or 70 g/L < Hb < 100 g/L accompanied by anemia symptoms, blood transfusion intervention was performed.

No intervention was administered for patients with abnormal albumin; 40 g/L > albumin > 30 g/L, however, those with albumin < 30 g/L received intravenous infusion of albumin.

Patient with abnormal serum potassium of < 3 mmol/L received intravenous potassium supplementation; whereas those with 3 mmol/L < serum potassium < 3.5 mmol/L received oral potassium supplementation.

### Surgical procedure

All operations were performed by two surgeons. Cardiopulmonary function, liver function, kidney function, and blood routine, among other tests were performed before surgery to rule out any surgical contraindications. The patients received an intravenous injection of 1.5 g tranexamic acid 30 min before the operation, intra-articular injection of 0.5 g tranexamic acid after the incision was sutured, and tourniquets during the operation. The laboratory test indicators such as blood routine, liver and kidney function, and electrolytes were reviewed on the second day after the operation, and the patients were encouraged to exercise on crutches 3 days after the operation. The surgical procedure was as follows: The surgical incision was made at 6–10 cm above the patella and was extended to 1–2 cm from the tibial tubercle. The medial 1/3 of the insertion point of the patellar ligament was stripped, the patella reversed laterally, and the anterior and posterior cruciate ligaments cut off. Next, the meniscus, hyperplastic synovium, and marginal osteophyte were removed. Thereafter, intramedullary or extramedullary positioning was achieved by installing an osteotomy guide, followed by osteotomy of the femur and tibia, insertion of the appropriate size joint prosthesis, and moving the knee joint to check for range of motion.

### Statistical analysis

Qualitative and quantitative variables were analyzed by chi-square test and independent sample *t*-test, respectively. The binary logistic regression model was used to identify risk factors associated with abnormal postoperative experimental indicators that require clinical intervention. Next, we evaluated the predictive value of risk factors and obtained cut-off values by generating receiver operative characteristic (ROC) curves. All data were analyzed using SPSS software version 26 (Inc., Chicago, IL), with data followed by *P* < 0.05 considered statistically significant.

## Results

### Laboratory test characteristics

Abnormal laboratory parameters were observed in 713 patients who underwent unilateral TKA. In summary, serum albumin (96.5%) was the main abnormal indicator among all laboratory tests after surgery, followed by hemoglobin (95.8%) and creatinine (45.2%) concentration. However, only a small proportion of postoperative patients with abnormal laboratory parameters received appropriate interventions. These included 8.1%, 9.9% and 3.4% patients with low hemoglobin, low albumin and hypokalemia, respectively (Table 2).

**Table 2:** Results of routine laboratory tests for patients undergoing TKA surgery

Laboratory Test (*n* = 713)	Abnormal Laboratory Test Result (n; %)	Postoperative Clinical Treatment Required (n; %)
Routine blood test		
Hemoglobin	684 (95.8%)	58 (8.1%)
Platelets	98 (13.7%)	0 (0)
Liver function test		
Alanine aminotransferase	85 (11.9%)	0 (0)
Aspartate aminotransferase	134 (18.8%)	0 (0)
Albumin	688 (96.5%)	71 (9.9%)
Renal function test		
Creatinine	323 (45.2%)	0 (0)
Blood urea nitrogen	135 (18.9%)	0 (0)
Inflammation indicators test		
Erythrocyte sedimentation rate	230 (32.2%)	0 (0)
C-reactive protein	129 (2.7%)	0 (0)
Electrolytes		
Serum sodium	73 (10.2%)	0 (0)
Serum potassium serum potassium	200 (28.0%)	24 (3.4%)
Serum calcium	295 (41.3%)	0 (0)

*TKA* total knee Arthroplasty

### Risk factors in patients who required postoperative clinical treatment

#### Risk factors in patients requiring postoperative blood transfusion

Univariate analysis revealed no statistically significant differences in gender, preoperative comorbidities, and body mass index (BMI) between patients with and without blood transfusion. Conversely, age (*p* = 0.001), operation time (*p* = 0.013) and intraoperative blood loss (*p* < 0.001) were significantly higher in patients who received postoperative transfusion relative to those who did not. On the other hand, patients in the transfusion group had significantly lower preoperative hemoglobin (*p* < 0.001) level compared with those in the non-transfusion group (Table 3). Results from binary logistic regression analysis showed that age (OR = 1.148, *P* < 0.001), intraoperative blood loss (OR = 1.008, *P* < 0.001), and preoperative hemoglobin (OR = 0.665, *P* < 0.001) were independent risk factors of postoperative blood transfusion in TKA patients (Table 4).

**Table 3:** Postoperative blood transfusion for patients with abnormal hemoglobin after TKA Surgery

Factor	Treatment Group (*n* = 58)	No Treatment Group (*n* = 626)	*P* value
Age (years)	74.45 ± 4.86	66.55 ± 10.86	0.001
Sex (n)			0.067
Male	20	148	
Female	38	478	
BMI (kg/m^2^)	22.37 ± 3.40	24.40 ± 4.02	0.495
Smoking: n(%)	19(32.8%)	15(2.4)	0.137
Alcohol use: n(%)	28(48.3)	252(40.3)	0.235
Diabetes mellitus:n(%)	5(8.6%)	71(11.3%)	0.504
High blood pressure: n(%)	23(39.75)	234 (37.4)	0.732
Preoperative hb level (g/L)	103.91 ± 4.34	125.05 ± 11.05	< 0.001
Estimated blood loss (mL)	426.88 ± 306.54	194.49 ± 147.62	< 0.001
Operative time (minutes)	170.90 ± 49.43	137.10 ± 41.32	0.013

*BMI* body mass index, *TKA* total knee Arthroplasty, *HB* Hemoglobin

**Table 4:** Risk factors for postoperative blood transfusion in patients undergoing TKA Surgery

Risk factor	odds ratio	95% confidence interval	*P* value
Age	1.148	1.068–1.234	< 0.001
Preoperative hb level	0.665	0.590–0.748	< 0.001
Estimated blood loss	1.008	1.005–1.011	< 0.001
Operative time	1.007	0.996–1.019	0.210

*Hb* Hemoglobin

#### Risk factors in patients requiring postoperative albumin supplementation

Univariate analysis results revealed significant differences between the supplemented albumin and non-supplemented groups in gender, smoking, drinking, preoperative albumin, intraoperative blood loss, and operation time (Table 5). Furthermore, preoperative albumin (OR = 0.700, *P* < 0.001), operation time (OR = 1.011, *P* < 0.001), and intraoperative blood loss (OR = 1.004, *P* = 0.037) were significant independent risk factors for postoperative albumin supplementation in TKA patients (Table 6).

**Table 5:** Clinical characteristics of patients who required postoperative albumin supplement

Factor	Treatment Group (*n* = 71)	No Treatment Group (*n* = 617)	*P* value
Age (years)	69.61 ± 12.02	67.08 ± 10.48	0.212
Sex (n)			< 0.001
Male	31	149	
Female	40	468	
BMI (kg/m^2^)	22.98 ± 3.29	24.35 ± 4.05	0.331
Smoking: n(%)	28(39.4%)	147(23.8%)	0.004
Alcohol use: n(%)	38(53.5%)	246(39.9%)	0.027
Diabetes mellitus:n(%)	6(8.5%)	71(11.5%)	0.439
High blood pressure: n(%)	22(31.0%)	238(38.6%)	0.212
Preoperative albumin (g/L)	36.74 ± 2.47	42.50 ± 3.80	< 0.001
Estimated blood loss (mL)	354.51 ± 274.90	196.66 ± 155.29	< 0.001
Operative time (minutes)	212.11 ± 23.36	131.12 ± 36.26	< 0.001

**Table 6:** Risk factors for patients requiring postoperative albumin supplementation

Risk factor	Odds ratio	95% confidence interval	*P* value
Sex	2.566	0.587–11.206	0.210
Smoking	0.711	0.162–3.128	0.652
Alcohol use	1.359	0.607–3.045	0.456
Estimated blood loss	1.004	1.003–1.006	0.037
Operative time	1.011	1.004–1.018	< 0.001
preoperative Albumin	0.700	0.620–0.791	< 0.001

#### Risk factors in patients requiring postoperative potassium supplementation

Univariate and binary logistic regression analysis results showed that BMI (OR = 1.191, *P* = 0.007) and preoperative serum potassium (OR = 0.019, *P* < 0.001) were significant independent risk factors of postoperative serum potassium supplementation in TKA patients (Table 7 and Table 8).

**Table 7:** Clinical characteristics of patients who required postoperative potassium supplement

Factor	Treatment Group (*n* = 24)	No Treatment Group (*n* = 176)	*P* value
Age (years)	69.29 ± 6.87	66.88 ± 11.64	0.218
Sex (n)			0.144
Male	2	42	
Female	22	134	
BMI (kg/m^2^)	20.82 ± 1.84	24.24 ± 4.39	0.006
Smoking: n(%)	4(%)	38()	0.578
Alcohol use: n(%)	10(5)	75()	0.930
Diabetes mellitus:n(%)	2(%)	13(%)	0.869
High blood pressure: n(%)	15(%)	74(%)	0.059
Preoperative Potassium(g/L)	3.66 ± 0.23	4.27 ± 0.42	0.02
Estimated blood loss (mL)	287.50 ± 218.82	215.45 ± 185.80	0.189
Operative time (minutes)	144.58 ± 53.79	139.29 ± 42.42	0.098

**Table 8:** Risk factors for patients requiring postoperative potassium supplementation

Risk factor	Odds ratio	95% confidence interval	*P* value
BMI	0.615	0.479–0.789	< 0.001
Preoperative Potassium	0.001	0.000–0.009	< 0.001

### Diagnostic accuracy of risk factors for predicting postoperative clinical treatment

The diagnostic value of risk factors for clinical intervention after TKA was determined from constructed ROC curves and cut-off values. A larger area under the curve (AUC) of the ROC implies higher prediction accuracy of the risk factors as shown in Table 9. In summary, preoperative hemoglobin level showed the highest accuracy in predicting blood transfusion (AUC = 0.933, *P* < 0.001).

**Table 9:** Cutoff values of risk factors for patients requiring postoperative clinical treatment

Treatment	Risk factors	Cut-off value	Sensitivity	Specificity	Auc	*P* value
Transfusion	Age	69.5	87.9%	61.3%	0.767	< 0.001
	Estimated blood loss	225	77.6%	75.7%	0.839	< 0.001
	Preoperative hb level	111.5	91.9%	86.2%	0.933	< 0.001
Albumin	Estimated blood loss	225	77.6%	75.7%	0.839	< 0.001
	Operative time	152.5	63.8%	69.6%	0.696	< 0.001
	preoperative Albumin	42.85	43.8%	74.1%	0.592	0.02
Potassium	BMI	21.89	73.9%	83.3%	0.793	0.04
	Preoperative potassium	3.68	93.8%	75%	0.912	0.028

*BMI* body mass index, *AUC* area under the curve

## Discussion

In recent years, medical workers have subjected many patients to excessive routine laboratory tests. This not only imposes a high financial burden on patients, but also unnecessary wastage of medical resources. To avoid this, researchers have questioned whether routine laboratory tests after surgery are necessary. Numerous studies have shown that routine postoperative laboratory tests are not needed because they lack clinical relevance [4, 13]. However, it is necessary for patients with risk factors to undergo postoperative laboratory testing [4, 14]. Other researchers have suggested that some unnecessary laboratory tests should not be carried out to reduce healthcare costs. In the present study, we aimed to verify the necessity for routine postoperative laboratory testing in patients undergoing primary unilateral total knee arthroplasty.

Laboratory tests play a crucial role in the diagnosis and monitoring the progress diseases. Gerald et al [15] reported that the more laboratory tests a patient is subjected to, the more likely it is that an abnormality will eventually be found. However, some laboratory tests are not necessary. Numerous studies have shown that routine postoperative laboratory tests are not required in most cases, except where risk factors are present [3, 4, 13–17]. For example, Li et al [14] showed that nearly 50% of patients who underwent high tibial osteotomy exhibited abnormal postoperative laboratory results, but less than 4% required clinical intervention, suggesting that routine postoperative serology is not required in a majority of patients. In this study, 95.8% of patients had abnormal postoperative hemoglobin levels, but only 8.1% of patients received clinical intervention. It was 28% probability of abnormal blood potassium after surgery, but only 3.4% of patients have been clinically intervened. Therefore, in most cases, patients do not need routine laboratory tests after receiving TKA surgery, except when risk factors are present. Statistical analysis of patients with abnormal laboratory test results who received clinical intervention revealed that age, intraoperative blood loss, and preoperative low hemoglobin were risk factors for postoperative anemia that will requiring intervention. Moreover, the operation time and preoperative albumin were found to be independent risk factors for postoperative albumin supplementation, whereas the body mass index (BMI) and preoperative hypokalemia were risk factors for postoperative potassium supplementation.

In another study, Dai et al [18] reported that age and low hemoglobin at admission were important risk factors for perioperative blood transfusion. Armin et al [19] retrospectively analyzed 8461 elderly patients, aged over 65 years and who underwent hip replacement in the United States in 2016. They found that old age and preoperative anemia were independent risk factors of postoperative blood transfusion. Moreover, Cao et al [20] retrospectively analyzed 414 and 1147 patients who underwent total hip arthroplasty and total knee arthroplasty, respectively. They reported that increased intraoperative blood loss was a risk factor of postoperative blood transfusion. In the present study, older age, low preoperative hemoglobin, and increased intraoperative blood loss were significant risk factors of postoperative blood transfusions, consistent with previous studies.

In another study, Wu et al [13] retrospectively analyzed 213 patients who underwent dance hip arthroplasty and found that a postoperative albumin incidence of 72.3%, of which 19.7% received clinical intervention. In the present study, we found that although the incidence of postoperative abnormal serum albumin was as high as 96.5%, only 9.9% of the patients received clinical intervention, which was lower than that reported by Wu et al [13]. In another study, Wu et al. [12] found that long operation time and low preoperative albumin levels were risk factors for postoperative albumin supplementation, which is consistent with our findings. Further, our results showed that increased intraoperative blood loss was also an important factor for albumin supplementation, as increased operative time increases intraoperative blood loss in patients. Li et al. [14] analyzed 482 patients who underwent high tibial osteotomy and found a low proportion of postoperative electrolyte abnormalities, including 3.5% of abnormal serum potassium. Preoperative serum potassium concentration below 3.45 mmol/L was an independent risk factor for postoperative potassium supplementation. In another study, Jordan et al. [10] retrospectively analyzed data from 213 patients who underwent shoulder arthroplasty and found that postoperative electrolyte abnormalities were strongly associated with lower BMI. Results of the present study showed that postoperative patients had a lower probability of electrolyte abnormalities, preoperative serum potassium concentration less than 3.68 mmol/l, and BMI less than 21.89 were important risk factors for postoperative potassium supplementation, consistent with the previous studies.

This study had some shortcomings. Firstly, this was a single-center retrospective study, with few studies, some with missing data. Some chronic medications are not taken into account. These may have introduced study bias to a certain extent. Secondly, there were differences among surgeons in the abnormal postoperative laboratory tests performed, which may also cause bias in the final results. Further studies, based on a multicenter study design, with a larger sample size, are needed to verify the effectiveness of these risk factors in predicting the need for clinical intervention in patients with abnormal laboratory tests after TKA.

## Conclusion

In summary, although most laboratory findings tend to be abnormal in patients undergoing primary TKA, most abnormalities are borderline and very few patients require further clinical intervention. Based on our results, we conclude that routine scheduling of postoperative laboratory tests after TKA surgery is not necessary. However, routine postoperative laboratory tests may be necessary for patients with established risk factors. For patients aged > 69.5 years, with an intraoperative blood loss > 225 ml, and preoperative hemoglobin < 111.5 g/l, we recommend elective complete blood count after operation. For patients with preoperative albumin < 42.85 g/l and operation time > 152.5 min, intraoperative albumin infusion can be considered. For patients with BMI < 21.89 kg/m^2^ and preoperative serum potassium < 3.68 g/l, we recommend basal metabolic group after operation. However, the above inferences need to be further validated with studies with larger sample sizes.

## Supplementary Information

**Additional file 1.** 

## Data Availability

All data generated or analysed during this study are included in this published article [and its supplementary information files].
